# Cricket protein-based film containing *Caralluma fimbriata* extract-based nanoparticles for preservation of cheddar cheese

**DOI:** 10.1016/j.ultsonch.2024.107167

**Published:** 2024-11-29

**Authors:** Aunzar Bashir Lone, Hina F. Bhat, Sunil Kumar, Abderrahmane Aït-Kaddour, Rana Muhammad Aadil, Abdo Hassoun, Zuhaib F. Bhat

**Affiliations:** a Division of Livestock Products Technology, SKUAST-J, Jammu, India; b Division of Animal Biotechnology, SKUAST-K, Kashmir, India; c Université Clermont Auvergne, INRAE, VetAgro Sup, UMRF, 15000 Aurillac, France; d Department of Food Technology, Faculty of Agroindustrial Technology, University of Padjadjaran, Sumedang 45363, Jawa Barat, Indonesia; e National Institute of Food Science and Technology, University of Agriculture, Faisalabad, Pakistan; f Sustainable AgriFoodtech Innovation & Research (SAFIR), Arras, France

**Keywords:** Cricket protein, Composite film, *C. fimbriata*, Silver nanoparticles, Cheddar cheese, Storage quality

## Abstract

A bioactive film was developed using cricket (*Acheta domestica*) protein (Cric-Prot) and *Caralluma fimbriata* extract-based nanoparticles [Car-Fim-NPs (0.0, 1.0, 2.0, and 3.0 % w/v)] to augment the storage stability and functional value of cheddar cheese (Ched-Chee). The Car-Fim-NPs were developed using an ultrasonication-mediated (20 kHz, 500 W, 10 min, pulse duration of 5/5 s on/off) green method. The addition of Car-Fim-NPs modified the film characteristics [density (%), WVTR (mg/mt^2^), transmittance (%), elongation at break (%), and colour (L*, a*, b*)] and enhanced its antioxidant [DPPH, ABTS and FRAP activities, total phenolic and flavonoid contents, and antioxidant release (%)] and antimicrobial (inhibitory halos against *E. coli* and *S. aureus*) potential. Application of the films containing Car-Fim-NPs (1.0–3.0 %) increased the antioxidant potential (DPPH, ABTS and FRAP activities), lipid (TBARS and free fatty acids) and protein (total carbonyl content) oxidative stability, and microbial quality (microbial counts) of the Ched-Chee during 90 days of storage. The sensory quality of Ched-Chee showed a significant increase after day 30 till the storage end. Gastrointestinal digestion simulation augmented the antioxidant potential of Ched-Chee. Overall, the results indicate the possibility of the use of Cric-Prot-based film containing Car-Fim-NPs to enhance the storage quality of Ched-Chee.

## 1 Introduction

Bioactive films developed using natural biopolymers are gaining the attention of researchers and consumers worldwide[1]. Different biopolymers have been used to develop bioactive and biodegradable packaging materials for food applications with a special focus on the valorisation of biopolymers extracted from agricultural wastes and side streams[1]. Studies have reported using carbohydrates, proteins and lipids extracted from plants, algae, fungi, and marine and terrestrial animals to develop packaging systems [2]. However, the use of insect proteins for developing food packaging materials and systems has received little to no attention. There are several countries where insects do not constitute a part of the human diet and can be easily diverted for developing alternative non-food-based products such as biodegradable packaging materials for food and non-food commodities. The use of insects for developing packaging materials can help divert several food and feed-grade biopolymers, such as maize protein and starch, which are used for developing biodegradable packaging materials and can otherwise be fed to animals or humans, especially in hunger-stricken third-world countries. The ever-expanding human and animal population demands the use of food and feed ingredients to be confined for edible purposes only. Further, the use of a vast and untapped resource for developing packaging materials can ease the environmental burden of petroleum-based materials which have polluted all the ecosystems on earth. There is a need to initiate extensive research in this area to develop successful packaging solutions and alternatives.

Among the various edible insects, crickets have a better social acceptability and are consumed in most parts of the world where insects are relished as food. The use of edible crickets as an alternative protein ingredient has been widely explored recently for the development of different cereal and animal protein-based food products for human consumption. Several bakery and chocolate-based products have been developed and some have already made it to the international markets including Europe, Canada and the USA. However, these products have acceptance issues in several countries including Western nations[3] suggesting the use of insect proteins for alternative purposes for full exploitation of this untapped resource. Compared to the other food animals, the crickets can be grown on farms in less time using limited resources and contain much higher levels of protein. These characteristics make crickets an ideal candidate for the development of biodegradable and alternative packaging systems and solutions. The present study used cricket protein to develop a bioactive film to enhance the stability of cheddar cheese (Ched-Chee) during storage. The *Caralluma fimbriata* plant extract-based nanoparticles (Car-Fim-NPs) were developed using a green method and incorporated in the film (0.0–3.0 %) to impart bioactive properties. The extract of *C. fimbriata* has been reported to have strong antioxidant and antimicrobial properties and contain several bioactive phytochemicals such as phenolic compounds, flavonoids, saponins, alkaloids, anthocyanins, coumarins, tannins/gallic tannins, diterpenes, quinones, terpenoids, anthraquinones, pregnane steroids, trigonelline, and glycosides [4].

## 2 Material and methods

### 2.1 Cric-Prot flour, *C. fimbriata* extract and bioactive agent

The Cric-Prot flour supplied by JR Unique Foods Ltd. (contained approximately 70 g protein, 20 g fat and 10 g carbohydrates per 100 g flour) was defatted using food-grade hexane (1:5 ratio, w/v) following the method elaborated by Lone et al. [5]. The flour was developed using 100 % *Acheta domestica* crickets farmed in a HACCP and FDA-approved factory in Thailand (Udon Thani) and contained no additives (artificial colours, preservatives or flavours). The films were prepared using this defatted Cric-Prot flour which was packaged under a vacuum and stored in a refrigerator (4 ± 1 °C) till used. The *C. fimbriata* extract manufactured by Nutranto Inc. (Haryana, India) from the aerial part of the plant and supplied by Amalth Lifecare Pvt Ltd (Haryana, India) was used for developing the bioactive agent for the Cric-Prot-based films. The extract dissolved in ethanol (85 % in water) was stirred for 2 h on a magnetic stirrer maintained at 50 °C and filtered through two different filter papers (Whatman No.1 and 42) separately. The filtrate was reduced in volume using a rotary vacuum-evaporator (35 ± 2 °C) and finally freeze-dried and converted to a powder form (Car-Fim-Ext) that was stored in a freezer (−20 °C). This powder was used for the development of Car-Fim-NPs for the films to add bioactive properties to them. Car-Fim-NPs were developed following a green method and using a Cole-Parmer ultrasonicator (WW-04711–45, U.S.A), the Car-Fim-Ext (5 %, w/v) and 1 mM aqueous solution of silver nitrate. The Car-Fim-Ext (10 ml) and silver nitrate solution (90 ml) were processed at 500 W and 20 kHz (5/5 s on/off) for 10 min and observed for a colour change on a magnetic stirrer (1 h). The formation of Car-Fim-NPs was indicated by a colour change initiated by the reduction of metal ions by the proteins and phytochemicals present in Car-Fim-Ext turning the pale-yellow solution to a reddish-brown. This was followed by a centrifugation step [12,000 rpm × 30 min (Refrigerated Centrifuge RH-152, UNILAB, India)] and the Car-Fim-NPs were obtained in a powder form after the freeze-drying process and were stored in a freezer (−20 °C) until used for the development of the films as a bioactive agent. The particle characteristics [average particle size (nm), zeta potential (mV), and polydispersity index] and confirmation of the Car-Fim-NPs synthesis were done using a Zetasizer Nano-ZS particle analyzer (Malvern, Worcestershire, UK) and by evaluating the absorption peak using a UV–Vis spectrophotometer which was found to be around 440 nm. The Car-Fim-NPs were subjected to evaluation for their antimicrobial and antioxidant potential by assessing FRAP (ferric reducing antioxidant power, µM TE/100g) and ABTS and DPPH radical scavenging activities (% inhibition) and determining total flavonoid and total phenolic contents in addition to inhibitory halos (mm) and minimum inhibitory concentration (MIC, mg/ml) against *Escherichia coli* and *Staphylococcus aureus* following the details elaborated in our previous paper [5].

### 2.2 Characterization and preparation of the Cric-Prot-based films

A solvent casting method of film formation was employed to develop the Cric-Prot-based films containing four levels of Car-Fim-NPs (0.0, 1.0, 2.0, and 3.0 % w/v)[6]. The final method (processing and ingredient levels) was chosen after a trial and error technique using different levels and conditions to obtain a film with desirable characteristics and optimum bioactive properties. The optimization data has been submitted to another journal for publication. The defatted Cric-Prot (0.6 g) was taken along with carrageenan (0.9 g) at a ratio of 60:40 in distilled water (100 ml), stirred using a glass rod and a plasticizer was added (14 % glycerol) and the solution was mixed again and kept on a magnetic stirrer (20 min at 80 °C) while constantly being stirred. Uniform layered films were developed by pouring uniform layers of the filmogenic solutions (60 °C) on glass plates which were dried in an air-drying oven (5 h at 55 °C). The Cric-Prot-based films were stored in a refrigerator for further analysis after being peeled from the glass surface and kept inside laminate pouches. The Cric-Prot-based films were subjected to characterization by evaluating their colour [lightness (L*), yellowness/blueness (b*), and redness/greenness (a*)], physicomechanical characteristics (density, thickness, water vapour transmission rate, elongation at break, transmittance, and moisture content) and antioxidant and antimicrobial potential (radical scavenging and ion-reducing activities, total phenolic and flavonoid contents and inhibitory halos against *E. coli* and *S. aureus*).

### 2.3 Ched-Chee and storage study

The Ched-Chee containing 374 kcal of energy, 30 percent fat, 25 percent protein, 1 percent carbohydrates and 5 percent ash (620 mg sodium) was purchased from a well-known brand in India namely Amul Pvt Ltd (Gujrat, India). The Cric-Prot-based films containing Car-Fim-NPs [0.0 (T_0_), 1.0 (T_1_), 2.0 (T_2_), and 3.0 % (T_3_)] were used to wrap the uniform samples (∼50 g cubes) of Ched-Chee which were placed in a refrigerator at 4 ± 1 °C for 90 days and compared with control Ched-Chee samples without any film. All the samples (with and without films) were packed within LDPE (low-density polyethylene) pouches. The Ched-Chee was analysed on different storage time points (days 0, 30, 60, and 90) for microbial and lipid stability, protein oxidation, physicochemical and digestion properties, and bioactivities (antimicrobial and antioxidant) to assess the impact of Cric-Prot-based films on quality and functional value of the cheese.

### 2.4 Bioactive potential (antioxidant and antimicrobial)

The Car-Fim-NPs, Cric-Prot-based films and Ched-Chee samples stored within the films were assessed for antioxidant potential by determining the total phenolic [expressed as µg Gallic acid equivalents (GAE)/mg and using the Folin-Ciocalteu method] and total flavonoid [expressed as µg Quercetin equivalents (QE)/mg and using the aluminium chloride colourimetric assay] contents and/or free radical and ion reducing capabilities by evaluating the ferric ion reduction (FRAP) capacity (µM TE/100 g) and DPPH and ABTS radical quenching activities (% inhibition) using calorimetric methods and following the details described by Lone et al. [5] and Noor et al. [6]. The Cric-Prot-based films were also assessed for antioxidant release % to express the release rate of phenolic compounds from Cric-Prot-based films using the method followed by Noor et al. [6]. The Car-Fim-NPs, Cric-Prot-based films and Ched-Chee samples were assessed for antimicrobial properties. The MIC (mg/ml) of Car-Fim-NPs was determined using a protocol (microdilution technique using a 96-well plate) described by Lone et al. [5]. The Car-Fim-NPs (1.0, 2.0 and 3.0 %) and Cric-Prot-based films (1.0, 2.0 and 3.0 %) were evaluated for inhibitory halos [disk agar diffusion tests using Muller Hinton agar (24 h incubation at 37 °C)] against both gram-positive and gram-negative bacteria (*S. aureus* and *E. coli,* 10^6^ cfu/ml). The Ched-Chee samples stored for 90 days within Cric-Prot-based films were enumerated (log_10_ cfu/g) for various microbial counts [yeast/moulds using potato dextrose agar, coliform using violet-red bile agar, psychrophilic using total plate agar and total plate using total plate agar] on regular storage time points (days 0, 30, 60 and 90) [5].

### 2.5 Characteristics of Cric-Prot-based films

The Cric-Prot-based films containing Car-Fim-NPs (0.0, 1.0, 2.0 and 3.0 %) were evaluated for colour [brightness (L*), yellowness/blueness (b*) and redness/greenness (a*) employing a Hunterlab colourimeter (U.S.A)] and various physicochemical and mechanical characteristics such as thickness (mm, digital micrometre)**,** transmittance (%, UV–Vis spectrophotometer), moisture content (%, hot air drier), water vapour transmission rate (mg/m^2^t, test cell with water), and density (g/ml, floatation method using heptane and CCl_4_) [6].

### 2.6 Lipid stability, protein oxidation, moisture content and digestion simulation

The methodologies elaborated by Lone et al. [5] for Ched-Chee were applied to determine the two most commonly used lipid stability parameters [free fatty acids (% oleic acid) and TBARS (mg MDA/kg)] and total carbonyl content (protein oxidation was measured as nmol/mg protein) of Ched-Chee samples during 90 days storage. The stored Ched-Chee samples were analysed for moisture content employing the gravimetric method described by Noor et al. [6] for cheese. The Ched-Chee samples from days 0 and 90 were subjected to digestion simulation (gastrointestinal phases) following details elaborated by Lone et al. [5] for cheese. This was done to evaluate the impact of digestion on the antioxidant potential of Ched-Chee. Ched-Chee samples (∼437.50 mg protein) were subjected to two phases of digestion [gastric using pepsin at pH 1.9 for 1 h followed by intestinal using pancreatin for 2 h (substrate: enzyme = 100: 1 w/w for both pepsin and pancreatin)] in plastic (polyvinyl) containers placed on magnetic multi-stirrer to maintain temperature (37 °C) and stirring (using stir bars or fleas) during digestion. Sodium phosphate buffer (0.1 M, pH 8.0) was used to change the pH after gastric digestion. The samples for analysis were taken after completion of the digestive simulation and were subjected to centrifugation for 15 min (4000×*g*) and clear supernatants were collected and analyzed (DPPH, ABTS and FRAP activities).

### 2.7 Sensory and texture profile analyses

The trained panels used to evaluate the Ched-Chee [3 replications for each time point (days 0, 30, 60, and 90)] comprised ten members (30–50 years old five males and five females) with years of experience with routine sensory testing of cheese and meat products. The participants provided written consent, followed all ethical guidelines and regulations and used an 8-point scale to evaluate the Ched-Chee samples (marked by 3-digit codes and randomized) for four significant attributes (flavour, body and texture, overall acceptability, and colour and appearance). The Ched-Chee was evaluated for texture profile analysis (firmness, springiness, adhesiveness, chewiness, cohesiveness, and resilience) using a texture analyser (a 25  kg load cell, probe of 5 cm diameter, 20 × 20 mm samples) imported from Japan (Shimadzu EZ-SX) on days 0 and 90 [6].

### 2.8 Statistical analysis

The results presented in the Tables and Figures (as means ± standard errors) were obtained after analysing data from various experiments (replicated six times, n = 6) using ANOVA [one-way ANOVA (film and particle characteristics) or two-way ANOVA (storage characteristics) using a General linear model at 0.05 significance level or repeated measurements ANOVA (sensory data, 0.05 significance level)] using SPSS-21. The individual pair of means were subjected to DMRT to determine the significance.

## 3 Results and Discussion

### 3.1 Characteristics of the film and Car-Fim-NPs

#### 3.1.1 Physico-mechanical characteristics of the film

The data relating to the physico-mechanical properties of Cric-Prot-based film is presented in Table 1. No effect (P > 0.05) of the addition of Car-Fim-NPs [1.0 % (T_1_), 2.0 % (T_2_), and 3.0 % (T_3_)] was observed on the thickness (mm), moisture content (%) and elongation at break (%) of Cric-Prot-based film. The addition of Car-Fim-NPs showed a significant (P < 0.05) increase in the density (g/ml) of Cric-Prot-based film which in turn resulted in an expected decrease in transmittance (%) and water vapour transmission rate (WVTR, mg/mt^2^). This increase in density might be due to the high density of Car-Fim-NPs which contains silver metal. The proteins and polysaccharides are generally hydrophilic and allow the interaction of water molecules with the film matrix. The hydroxyl and amino groups in proteins act as the binding sites for water molecules, facilitating their permeability through the film matrix. The presence of Car-Fim-NPs in the film matrix can impede the free interaction of water molecules with binding sites in the film matrix and reduce the WVTR. Further, crosslinking of various phytochemicals with hydroxyl and amino groups also limits the interaction of water molecules with these binding sites and reduces the transmission of water vapours. The addition of plant extract-based silver nanoparticles has been found to increase the density and decrease the transmittance (%) and WVTR of protein-based bioactive films [6]. Similar results were reported by Gao et al. [7] on the incorporation of cinnamaldehyde in whey protein and hydroxypropyl methylcellulose-based edible films. A decrease in the WVTR has been reported for chitosan and hydroxyethyl cellulose-based edible films on incorporation of olive leaf extract and titanium dioxide nanoparticles [8].

**Table 1:** Characteristics of the Cric-Prot-based film and Car-Fim-NPs.

**Car-Fim-NPs**					
Parameters				Mean ± S.E	
Total phenolic content (µg GAE/mg)				78.62 ± 0.14	
Total flavonoid content (µg QE/mg)				24.65 ± 0.11	
DPPH (% inhibition)				76.82 ± 0.32	
ABTS (% inhibition)				86.42 ± 0.43	
FRAP (µM TE/100 g)				19.93 ± 0.24	
Average particle size (nm)				18.23 ± 2.14	
Maximum absorption peak (nm)				440	
Zeta potential (mV)				−18.42 ± 0.48	
Polydispersity index (PDI)				0.324	
MIC (minimum inhibitory concentration, mg/ml)		*S. aureus*		3.0	
		*E. coli*		10.0	
Inhibitory halos (mm)		*S. aureus*	1.0 %	14.25 ± 0.14^C^	
			2.0 %	16.48 ± 0.18^B^	
			3.0 %	17.65 ± 0.11^A^	
		*E. coli*	1.0 %	12.45 ± 0.12^A^	
			2.0 %	13.55 ± 0.24^B^	
			3.0 %	15.43 ± 0.22^A^	

**Cric-Prot-based film**					
**Parameters**		**T_0_ (0.0 %)**	**T_1_ (1.0 %)**	**T_2_ (2.0 %)**	**T_3_ (3.0 %)**
Thickness (mm)		0.147 ± 0.04^a^	0.147 ± 0.05^a^	0.148 ± 0.04^a^	0.148 ± 0.06^a^
Moisture content (%)		31.80 ± 0.24^a^	31.55 ± 0.24^a^	31.28 ± 0.32^a^	30.91 ± 0.28^a^
Density (g/ml)		0.83 ± 0.003^a^	0.85 ± 0.004^b^	0.87 ± 0.004^c^	0.89 ± 0.003^d^
WVTR (mg/mt^2^)		1.91 ± 0.002^a^	1.88 ± 0.004^b^	1.86 ± 0.005^c^	1.84 ± 0.003^d^
Transmittance (%)		85.72 ± 0.33^a^	83.04 ± 0.34^b^	81.96 ± 0.34^c^	79.85 ± 0.34^d^
Elongation at break (%)		48.25 ± 1.35^a^	48.04 ± 1.20^a^	47.80 ± 1.05^a^	47.60 ± 1.10^a^
Lightness (L*)		74.40 ± 0.22^a^	72.01 ± 0.27^b^	70.78 ± 0.34^c^	68.80 ± 0.32^d^
Redness (a*)		0.81 ± 0.004^a^	0.83 ± 0.002^b^	0.85 ± 0.003^c^	0.87 ± 0.003^d^
Yellowness (b*)		3.11 ± 0.04^a^	3.34 ± 0.03^b^	3.56 ± 0.04^c^	3.73 ± 0.03^d^
Total phenolic content (µg GAE/mg)		0.82 ± 0.04^d^	19.98 ± 0.18^c^	36.90 ± 0.12^b^	51.14 ± 0.26^a^
Total flavonoid content (µg QE/mg)		1.02 ± 0.04^d^	22.12 ± 0.62^c^	40.14 ± 0.38^b^	56.25 ± 0.12^a^
DPPH (% inhibition)		1.24 ± 0.01^d^	32.12 ± 0.42^c^	38.24 ± 0.22^b^	42.19 ± 0.44^a^
ABTS (% inhibition)		2.23 ± 0.04^d^	37.50 ± 0.13^c^	44.13 ± 0.24^b^	48.12 ± 0.42^a^
FRAP (µM TE/100 g)		0.88 ± 0.03^d^	15 ± 0.16^c^	17 ± 0.22^b^	19 ± 0.35^a^
Antioxidant release (%)		−	79.65 ± 1.30^b^	81.36 ± 1.15^b^	85.44 ± 1.05^a^
Inhibitory halos (mm)	*S. aureus*	0	12.25 ± 0.13^c^	13.40 ± 0.22^b^	14.54 ± 0.18^a^
	*E. coli*	0	11.40 ± 0.24^c^	12.38 ± 0.20^b^	14.45 ± 0.25^a^

Mean ± SE with different superscripts in a row (lower case alphabet) or column (upper case alphabet) differ significantly (P < 0.05), n = 6 for each treatment.

T_0_ (0.0 %) = film without any Car-Fim-NPs, T_1_ (1.0 %) = film with 1.0 % of the Car-Fim-NPs, T_2_ (2.0 %) = film with 2.0 % of the Car-Fim-NPs, T_3_ (3.0 %) = film with 3.0 % of the Car-Fim-NPs.

WVTR = water vapour transmission rate.

The high transmittance (transparency) of a packaging film is generally preferred by consumers due to the high visibility of packaged products that can influence the willingness to purchase [9]. High density and low transmittance can lower the exposure of the food to light including UV which has the potential to catalyse lipid oxidation and deteriorate the sensory quality. The protein-based film matrices are more efficient in retaining UV light [10]. A reduction in WVTR will reduce water availability, microbial growth and other adverse food reactions, such as browning and oxidation reactions, which generally reduce the sensory and food quality [11].

#### 3.1.2 Colour of the film and bioactive properties (film and Car-Fim-NPs)

The data relating to the colour and bioactive properties of Cric-Prot-based film is presented in Table 1. The addition of Car-Fim-NPs significantly (P < 0.05) increased the redness (a*) and yellowness (b*) and decreased the lightness (L*) of the Cric-Prot-based film. This increase in redness and yellowness of the Cric-Prot-based film may be attributed to various pigments and colourful compounds present in *C. fimbriata* extract which can also reduce the transmission of light through the film. A similar decrease in the L* values and increase in b* has been reported for chitosan films incorporated with *Saccharina latissimi* extract[12].

The addition of Car-Fim-NPs (1.0–3.0 %) showed a significant (P < 0.05) increase in the total phenolic (µg GAE/mg) and total flavonoid (µg QE/mg) contents of Cric-Prot-based film which in turn resulted in a significant (P < 0.05) increase in antioxidant potential [DPPH and ABTS radical scavenging (% inhibition) and ferric ion reduction (FRAP)] of the Cric-Prot-based film. This significant increase in the antioxidant potential of the Cric-Prot-based film might be attributed to the high total phenolic and flavonoid contents of Car-Fim-NPs (78.62 ± 0.14 µg GAE/mg and 24.65 ± 0.11 µg QE/mg, respectively). Various phytochemicals with strong antioxidant properties have been reported in *C. fimbriata* extract such as phenolic compounds, saponins, alkaloids, anthocyanins, anthraquinones, and glycosides [4]. This high total phenolic and flavonoid contents of Car-Fim-NPs reflected in their high DPPH, ABTS, and FRAP activities [76.82 ± 0.32 (% inhibition), 86.42 ± 0.43 (% inhibition), and 19.93 ± 0.24 (µM TE/100 g), respectively]. A recent study[13] has reported strong antioxidant potential (total phenolic content and DPPH and FRAP activities) of *C. fimbriata* powder (44.17 μgFe/g, 68.75 μgFe/g, and 800.81 μgFe/g, respectively). An effect of the concentration was also recorded in our study and the highest values for total phenolic and flavonoid contents and DPPH, ABTS, and FRAP activities were found for the Cric-Prot-based film containing the highest concentration of Car-Fim-NPs. This increasing pattern (P < 0.05) was also found for antioxidant release (%) from the film with the increasing concentration of Car-Fim-NPs. Flórez et al. [12] reported a similar increase in the antioxidant potential (DPPH and ABTS) of chitosan films incorporated with *Saccharina latissimi* extract. Recent studies have reported strong antioxidant properties for gold and silver nanoparticles developed using green synthesis methods [14], [15].

An ultrasound-mediated process (20 kHz, 500 W, 10 min, pulse duration of 5/5 s on/off) was used to produce the Car-Fim-NPs with an average particle size of 18.23 ± 2.14 nm, zeta potential of −18.42 ± 0.48, a polydispersity index of 0.324 and showed plasmon resonance (UV–Vis spectroscopy) at 440 nm (indicated by yellow–brown colour). While developing the silver nanoparticles using *C. fimbriata* plant extract by a green method, Pande et al. [16] reported an average particle size of 18 nm and plasmon resonance (UV–Vis spectroscopy) at 452 nm. When excited by light at specific wavelengths, the conduction electrons on the surface of silver undergo a collective oscillation called surface plasmon resonance and is generally found in the range of 400–500 nm. Haseena et al. [17] found a particle size of 25 nm for Fe_2_O_3_ nanoclusters developed using *C. fimbriata* as a reducing agent. Small-sized nanoparticles are more effective in imparting bioactive properties to the films by accommodating more phytochemicals and due to better release rates of the films for bioactive molecules.

The Cric-Prot-based film showed good antimicrobial properties as indicated by the zones of inhibition against *E. coli* and *S. aureus*. This antimicrobial potential of the Cric-Prot-based film might be attributed to Car-Fim-NPs which showed good antimicrobial properties (inhibitory halos and MIC) against *E. coli* and *S. aureus*. The inhibitory halos (mm) of the Cric-Prot-based film showed a significant (P < 0.05) increase with the increasing concentration of Car-Fim-NPs against both *E. coli* and *S. aureus.* The *C. fimbriata* plant extract contains several antimicrobial phytochemicals, such as tannins, phenols, glycosides, alkaloids, terpenoids, flavonoids, and steroids [18], and has reported antimicrobial activity against several microbes [18], [19], [20]. While studying the antimicrobial properties of silver nanoparticles developed using aqueous stem extract of *C. fimbriata*, Packialakshmi & Naziya [20] reported strong antimicrobial properties for the developed nanoparticles against both Gram-positive and Gram-negative bacteria. The zones of inhibition (mm) found against *S. aureus*, *B. subtilis*, *Proteus* sp., *Klebsiella* sp., *Staphylococcus epidermidis*, and *E. coli* were 16, 15, 18, 14, 14, and 15, respectively. This study [20] found several compounds in the *C. fimbriata* extract such as halogens, alcohols, esters, carboxylic acids, amides, ketones, alkynes, alkanes, acid anhydrides, alkenes, nitro compounds, aldehydes, lactones, amino acids, and acid halides. Haseena et al. [17] have reported strong antimicrobial properties of Fe_2_O_3_ nanoparticles synthesized using *C. fimbriata* extract against *E. coli* [zones of inhibition of 20 mm (75 µg/ml)]. An increase in the antimicrobial properties of chitosan and hydroxyethyl cellulose-based edible films has been reported by El-Sayed et al. [8] upon incorporation with olive leaf extract and titanium dioxide nanoparticles as indicated by the zones of inhibition against several microbes.

### 3.2 Effect of Cric-Prot-based film on the antioxidant potential of Ched-Chee

**Fig. 1:**
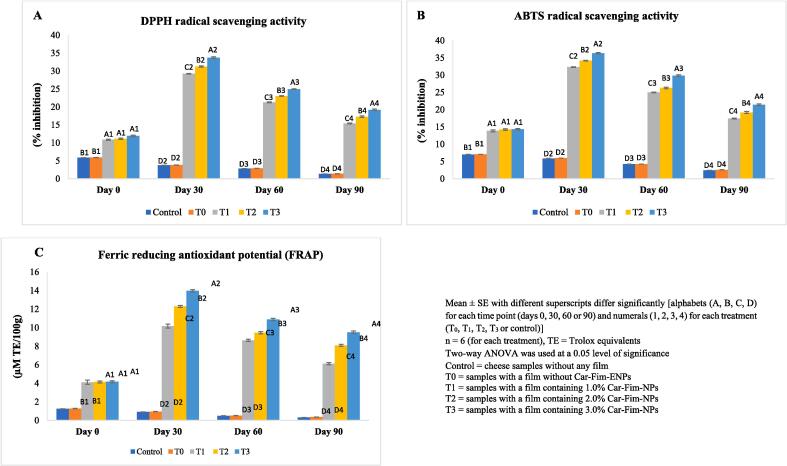
Effect of Cric-Prot-based film incorporated with Car-Fim-NPs on the antioxidant potential of cheddar cheese. Mean ± SE with different superscripts differ significantly [alphabets (A, B, C, D) for each time point (days 0, 30, 60 or 90) and numerals (1, 2, 3, 4) for each treatment (T_0_, T_1_, T_2_, T_3_ or control)]. n = 6 (for each treatment), TE = Trolox equivalents. Two-way ANOVA was used at a 0.05 level of significance. Control = cheese samples without any film. T0 = samples with a film without Car-Fim-ENPs. T1 = samples with a film containing 1.0 % Car-Fim-NPs. T2 = samples with a film containing 2.0 % Car-Fim-NPs. T3 = samples with a film containing 3.0 % Car-Fim-NPs.

The data relating to the antioxidant potential (DPPH, ABTS and FRAP) of Ched-Chee is presented in Fig. 1 (A-C). A significant (P < 0.05) positive effect of Cric-Prot-based film was found on the antioxidant potential of Ched-Chee during storage. The Ched-Chee samples packed within the Cric-Prot-based films containing Car-Fim-NPs [1.0 % (T_1_), 2.0 % (T_2_), and 3.0 % (T_3_)] showed significant (P < 0.05) higher values for DPPH, ABTS and FRAP compared to the control (without film) and samples packed within T_0_ films (0 % Car-Fim-NPs) during entire storage. A significant (P < 0.05) positive effect of the concentration was also found and the highest values (DPPH, ABTS, and FRAP) were found for the Ched-Chee packed within T_3_ films containing the highest concentration of Car-Fim-NPs (3.0 %) from days 30 to 90. This positive effect of Cric-Prot-based film on the antioxidant potential of Ched-Chee during storage may be attributed to the phytochemicals of *C. fimbriata* with antioxidant properties leaching out from the film matrix [4]. Flórez et al. [12] observed an increased antioxidant potential (DPPH and ABTS activities) of Havarti cheese packaged with chitosan film containing *Saccharina latissimi* extract compared to the control samples packaged with chitosan films*.* A decreasing trend in the DPPH activity of Havarti cheese samples whereas an increasing pattern in the ABTS activity was found with increasing storage time (45 days). Our results showed an initial increase in the antioxidant activities (DPPH, ABTS and FRAP) of Ched-Chee till day 30 followed by a decrease till the end of the storage. Studies have reported an initial increase in the antioxidant potential (DPPH, ABTS and FRAP) of Ched-Chee packed within bioactive films followed by a decrease towards the storage end [6]. The bioactive molecules get released from a film at an increasing rate during the first few days of storage until an equilibrium is reached, thereby increasing the antioxidant activity of cheese. This is followed by more and more quenching of bioactive molecules to reduce lipid and protein oxidation towards the end of the storage, resulting in a decline in the antioxidant activity of cheese.

### 3.3 Effect of Cric-Prot-based film on lipid stability and protein oxidation of Ched-Chee

**Fig. 2:**
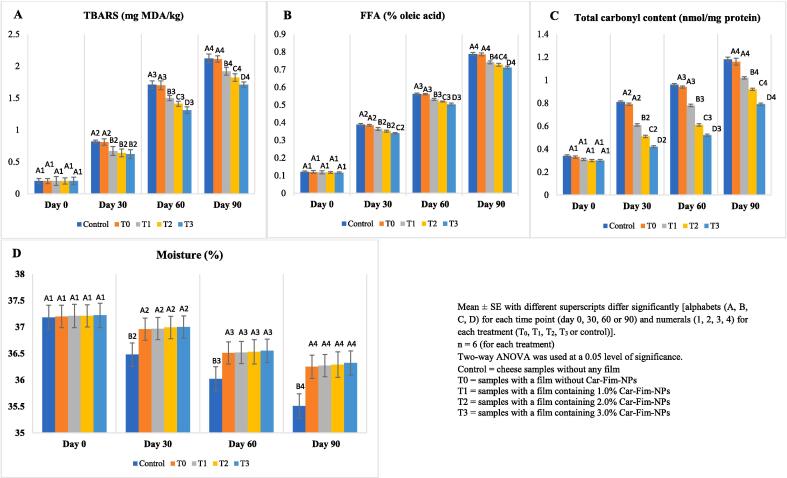
Effect of Cric-Prot-based film incorporated with Car-Fim-NPs on the antioxidant potential of cheddar cheese. Mean ± SE with different superscripts differ significantly [alphabets (A, B, C, D) for each time point (day 0, 30, 60 or 90) and numerals (1, 2, 3, 4) for each treatment (T_0_, T_1_, T_2_, T_3_ or control)]. n = 6 (for each treatment). Two-way ANOVA was used at a 0.05 level of significance. Control = cheese samples without any film. T0 = samples with a film without Car-Fim-NPs. T1 = samples with a film containing 1.0 % Car-Fim-NPs. T2 = samples with a film containing 2.0 % Car-Fim-NPs. T3 = samples with a film containing 3.0 % Car-Fim-NPs.

The data related to lipid stability [TBARS (mg MDA/kg) and FFA (% oleic acid)] and protein oxidation [total carbonyl content (nmol/mg protein)] of Ched-Chee during storage is presented in Fig. 2 (A-C). A significant (P < 0.05) positive effect of Cric-Prot-based film was found on the lipid stability of Ched-Chee samples from days 30 to 90 of storage. While both TBARS and FFA values of Ched-Chee showed a significant increase with storage time, the Ched-Chee samples packed within the Cric-Prot-based films containing Car-Fim-NPs [1.0 % (T_1_), 2.0 % (T_2_), and 3.0 % (T_3_)] showed significantly (P < 0.05) lower values compared to the control and samples packed within T_0_ films. A significant (P < 0.05) positive effect of the concentration of the Car-Fim-NPs was also found on the lipid stability of Ched-Chee on days 60 and 90 and the lowest values for TBARS and FFA were recorded for the T_3_ films containing the highest concentration of Car-Fim-NPs. The lipid oxidation (catalysed by oxygen and metal ions) and lipolysis induced by the enzymes (microbial and natural lipases) within Ched-Chee during storage results in an increase in TBARS and FFA values. The bioactive phytochemicals with strong antioxidant properties, such as phenolic compounds, flavonoids, saponins, alkaloids, anthocyanins, anthraquinones, and glycosides, are present in the extract of *C. fimbriata* [4]and may be responsible for the positive impact of the Cric-Prot-based film on the lipid stability of Ched-Chee during storage.

A significant (P < 0.05) positive effect of Cric-Prot-based film was also found on the protein oxidation of Ched-Chee samples from days 30 to 90. The Ched-Chee samples packed within the Cric-Prot-based films containing Car-Fim-NPs [1.0 % (T_1_), 2.0 % (T_2_), and 3.0 % (T_3_)] showed significant (P < 0.05) lower values compared to the control and samples packed within T_0_ films. A significant (P < 0.05) positive effect of the concentration of the Car-Fim-NPs was also found and the lowest carbonyl values were recorded for the Ched-Chee samples packed within the T_3_ films on days 30 to 90. The phytochemicals with antioxidant properties leaching out from the matrix of Cric-Prot-based films can scavenge free radicals and reduce lipid and protein oxidation in Ched-Chee samples during storage. Both these processes have the potential to adversely affect the nutritive and sensory characteristics of the Ched-Chee [6]. Flórez et al. [12] have reported a significant positive effect of a chitosan film containing an aqueous extract of Sugar kelp *Saccharina latissimi* on the lipid stability of Havarti cheese. The cheese samples packaged within the film showed a 52.80 % reduction in lipid oxidation (MDA/kg) after 45 days of refrigerated storage compared to the chitosan films (control). A significant positive effect of an edible coating of whey protein and nanoclay biocomposite containing *Thymus fedtschenkoi* essential oil (1.0 %) and resveratrol (0.001 %) has been reported on the lipid oxidation (TBARS and peroxide values) of liqvan cheese during storage [21]. This study also reported a significant positive effect of the film on the protein oxidation (carbonyl content) of liqvan cheese during storage and demonstrated that the addition of polyphenolic compounds to the edible coatings can reduce the synthesis of carbonyl compounds in cheese during storage. A significant decrease in the FFA values has been reported for sour cream packaged within chitosan and hydroxyethyl cellulose-based edible films incorporated with olive leaf extract and titanium dioxide nanoparticles [8].

### 3.4 Effect of Cric-Prot-based film on the moisture content of Ched-Chee

The data related to the moisture content of Ched-Chee during storage is presented in Fig. 2(D). A significant (P < 0.05) positive effect of Cric-Prot-based film was found on the moisture content of Ched-Chee samples from days 30 to 90 of storage. The Ched-Chee samples packed within the Cric-Prot-based films with or without Car-Fim-NPs (T_0_, T_1_, T_2_, and T_3_) showed significantly (P < 0.05) higher moisture content compared to the control samples without any film. Studies have reported a positive effect of the physical barriers, such as edible films, on the moisture retention of Ched-Chee during storage [6].

### 3.5 Effect of Cric-Prot-based film on digestion characteristics of Ched-Chee

**Fig. 3:**
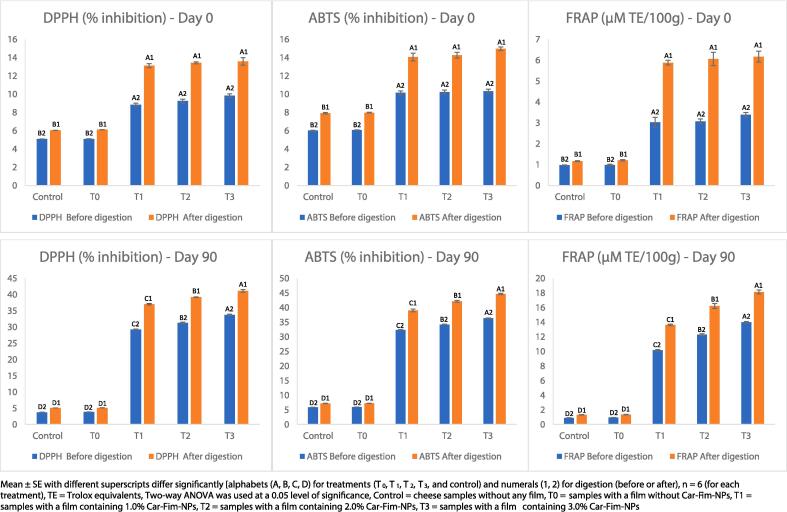
Effect of *in vitro* gastrointestinal digestion on the antioxidant potential of the cheddar cheese. Mean ± SE with different superscripts differ significantly [alphabets (A, B, C, D) for treatments (T_0_, T_1_, T_2_, T_3_, and control) and numerals (1, 2) for digestion (before or after), n = 6 (for each treatment), TE = Trolox equivalents, Two-way ANOVA was used at a 0.05 level of significance, Control = cheese samples without any film, T0 = samples with a film without Car-Fim-NPs, T1 = samples with a film containing 1.0 % Car-Fim-NPs, T2 = samples with a film containing 2.0 % Car-Fim-NPs, T3 = samples with a film containing 3.0 % Car-Fim-NPs.

The data relating to *in vitro* gastrointestinal digestion is presented in Fig. 3. The Ched-Chee samples packed within the Cric-Prot-based films were subjected to gastrointestinal digestion simulation on days 0 and 90 and the digested samples were evaluated for antioxidant properties (DPPH and ABTS radical scavenging activities and FRAP). A significant (P < 0.05) positive effect of Cric-Prot-based film was found on the antioxidant potential of digested Ched-Chee samples on both day 0 and day 90. The Ched-Chee samples packed within the Cric-Prot-based films containing Car-Fim-NPs (T_1_, T_2_, and T_3_) showed significantly (P < 0.05) higher values for all the parameters (DPPH, ABTS, and FRAP activities) before and after digestion compared to the control and samples packed within T_0_ films. This positive effect on the antioxidant potential of stored and digested Ched-Chee samples may be attributed to the release of bioactive molecules from Cric-Prot-based film. This indicates a positive impact of the film on the consumers’ health as the bioactive molecules are released from the film matrix and during the digestion of Ched-Chee. A positive effect of the edible films containing bioactive molecules (2 % *C. roseus* leaf extract-based silver nanoparticles) has been reported on the antioxidant potential of stored Ched-Chee samples after gastrointestinal digestion [6]. Similar results have also been reported by Jatav et al. [22] for Indian cottage cheese packed within the chitosan films containing pineapple peel extract (1, 2 and 3 %) while measuring the release kinetics of polyphenols from the film matrix and bioaccessibility of polyphenols during digestion. However, in contrast to our study, the cumulative release and bioaccessibility of polyphenols showed a negative relation with the concentration of the extract. This was attributed to various transformations that polyphenols undergo during the digestion process (hydrolysis oxidation, epimerization, and degradation) and the polyphenol interactions (such as ionic interactions and hydrogen bonding) with dietary and film components.

### 3.6 Effect of Cric-Prot-based film on the microbial quality of Ched-Chee

**Fig. 4:**
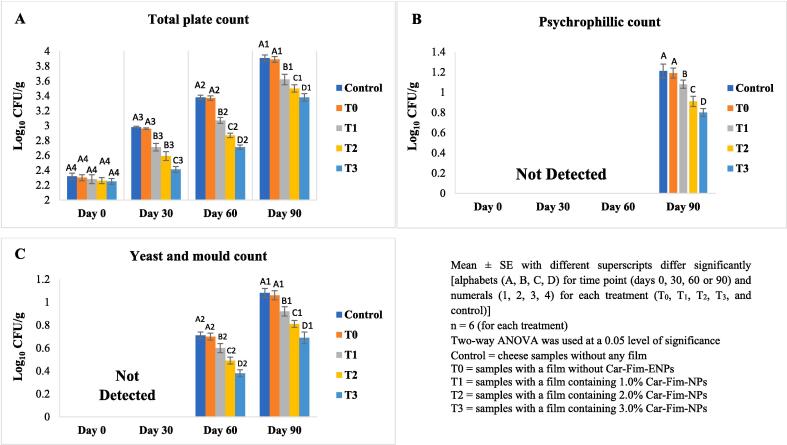
Effect of Cric-Prot-based film on the microbiological quality of cheddar cheese. Mean ± SE with different superscripts differ significantly (a, b), n = 10 (for each treatment), Two-way ANOVA was used at a 0.05 level of significance, Control = cheese samples stored without any film, T0 = cheese samples with a film containing 0 % NPs, T1 = cheese samples with a film containing 1 % NPs, T2 = cheese samples with a film containing 2 % NPs, T3 = cheese samples with a film containing 3 % NPs.

The data related to microbiological characteristics (total plate, psychrophilic, and yeast/mould counts) of Ched-Chee during storage is presented in Fig. 4 (A-C). A significant (P < 0.05) positive effect of Cric-Prot-based film was found on the microbial quality of Ched-Chee samples from days 30 to 90 of storage. The Ched-Chee samples packed within the Cric-Prot-based films containing Car-Fim-NPs (T_1_, T_2_, and T_3_) showed significantly (P < 0.05) lower total plate counts (log_10_ CFU/g) compared to the control and samples packed within T_0_ films. The lowest counts were observed for the samples packed within the T_3_ films. The yeast/moulds and psychrophiles were detected on days 60 and 90, respectively, and showed similar patterns. The Ched-Chee samples packed within the Cric-Prot-based films (T_1_, T_2_, and T_3_) showed significantly (P < 0.05) lower counts (log_10_ CFU/g) compared to the control and T_0_ films. The *C. fimbriata* plant extract contains several antimicrobial phytochemicals, such as tannins, phenols, glycosides, alkaloids, terpenoids, flavonoids, and steroids [18], and has reported antimicrobial activity against several microbes, such as *E. coli, Streptococcus epidermidis, Streptococcus mutans, Streptococcus sanguis, Bacillus subtilis, Enterococcus faecalis, Lactobacillus acidophilus*, *Pseudomonas aeruginosa, Staphylococcus aureus, Klebsiella, Proteus,* and Coliforms [18], [19], [20]. A significant positive effect of whey protein and nanoclay biocomposite-based coating containing *T. fedtschenkoi* essential oil (1.0 %) and resveratrol (0.001 %) has been reported on total mesophilic bacteria (decrease of more than 2 logarithms on day 42) and yeasts and moulds (decrease of more than 2 logarithms on day 42) of liqvan cheese during refrigerated storage [21]. A similar antimicrobial effect of nanocellulose films containing halloysite nanotubes encapsulated with *Zataria multiflora* essential oil has been reported on *Escherichia coli* inoculated white cheese with a maximum observed reduction of 1.6 log CFU/g during 12 days [23].

### 3.7 Effect of Cric-Prot-based film on the sensory and textural analysis of Ched-Chee

**Fig. 5:**
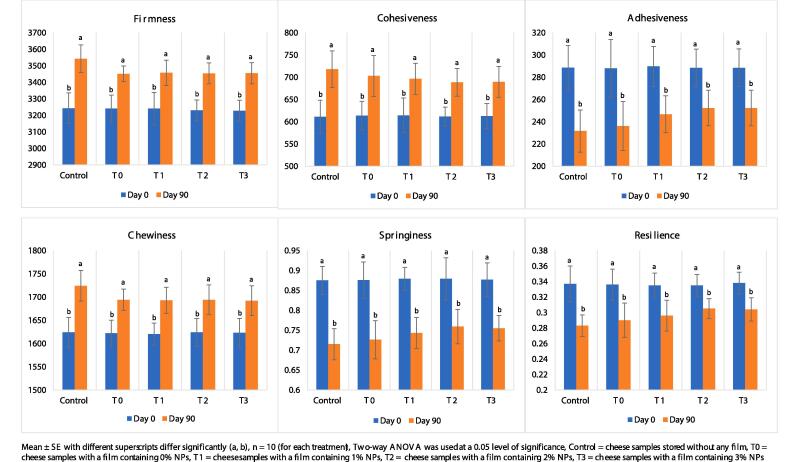
Effect of Cric-Prot-based film incorporated with Car-Fim-NPs on texture profile analysis of the cheddar cheese. Mean ± SE with different superscripts differ significantly [alphabets (A, B, C, D) for each time point (day 0, 30, 60 or 90) and numerals (1, 2, 3, 4) for each treatment (T_0_, T_1_, T_2_, T_3_ or control)], Repeated measurements ANOVA was used at a 0.05 level of significance, 10 panellists performed the sensory evaluation thrice for each treatment at each time point (days 0, 30, 60, and 90) using an 8-point descriptive scale (1 denoted ‘disliked extremely’ and 8 denoted ‘liked extremely’), Control = cheese samples without any film, T0 = samples with a film containing 0.0 % Car-Fim-NPs, T1 = samples with a film containing 1.0 % Car-Fim-NPs, T2 = samples with a film containing 2.0 % Car-Fim-NPs, T3 = samples with a film containing 3.0 % Car-Fim-NPs.

**Fig. 6:**
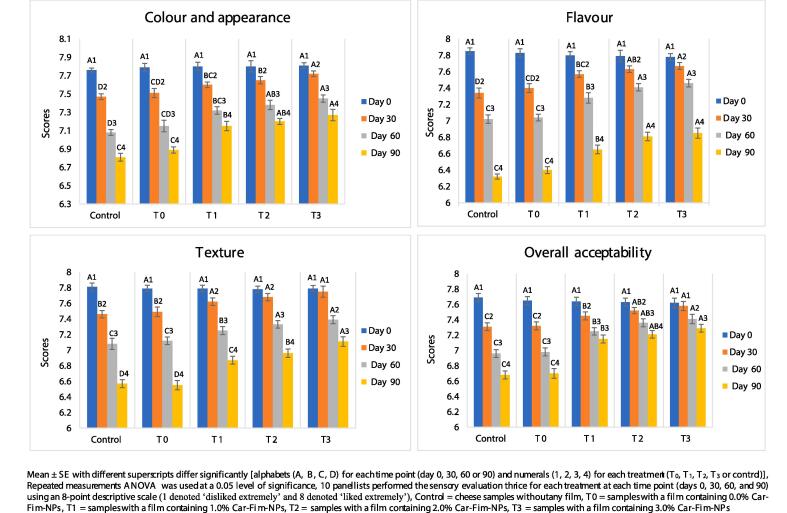
Effect of Cric-Prot-based film incorporated with Car-Fim-NPs on sensory quality of the cheddar cheese.

The data related to the textural and sensory analysis of Ched-Chee during storage is presented in Fig. 5, Fig. 6. A significant (P < 0.05) positive effect of Cric-Prot-based film was found on the sensory quality of Ched-Chee samples from days 30 to 90 of storage. The Ched-Chee samples packed within the Cric-Prot-based films containing Car-Fim-NPs (T_1_, T_2_, and T_3_) showed significantly (P < 0.05) higher scores for all the attributes (appearance and colour, texture, overall acceptability, and flavour) compared to the control and samples packed within T_0_ films. The Cric-Prot-based films significantly reduced the lipid oxidation and microbial load of Ched-Chee samples during storage and thus retained better sensorial characteristics. Both lipid oxidation and microbial spoilage deteriorate the sensory quality of food products during storage. A significant positive effect of whey protein and nanoclay biocomposite-based coating containing *T. fedtschenkoi* essential oil (1.0 %) and resveratrol (0.001 %) has been reported on the sensory quality of liqvan cheese during refrigerated storage [21]. Similar positive effects of bioactive films have been reported by Jatav et al. [22] and Noor et al., [6] on the sensory quality of Indian cottage cheese and Ched-Chee during refrigerated storage. No significant (P > 0.05) impact of the Cric-Prot-based film was recorded on the texture profile analysis of Ched-Chee during storage and might be attributed to the fact that all the samples (with and without film) were packed within the LDPE pouches during storage.

## 4 Conclusions

A Cric-Prot-based film was prepared using Car-Fim-NPs [0.0 (T_0_), 1.0 (T_1_), 2.0 (T_2_), and 3.0 % (T_3_)] as a bioactive agent to augment the storage stability of Ched-Chee. The Ched-Chee samples packed within the Cric-Prot-based films containing Car-Fim-NPs (T_1_, T_2_, and T_3_) exhibited significantly lower lipid oxidation and microbial spoilage during refrigerated storage compared to the control and samples packed within T_0_ films. This positive effect of the Cric-Prot-based film was reflected in the sensory quality of Ched-Chee, attaining higher scores for all sensory attributes after day 30. The Cric-Prot-based film containing Car-Fim-NPs can be successfully used to enhance the storage stability of Ched-Chee. A positive impact of the digestion was found on the antioxidant potential of Ched-Chee. The film should be evaluated for other food products. Future studies should focus on developing new insect-based materials to replace the plastic films.

## CRediT authorship contribution statement

**Aunzar Bashir Lone:** Methodology, Investigation, Formal analysis. **Hina F. Bhat:** Conceptualization, Writing – review & editing, Resources. **Sunil Kumar:** Supervision, Methodology, Data curation. **Abderrahmane Aït-Kaddour:** Writing – review & editing. **Rana Muhammad Aadil:** Writing – review & editing, Visualization, Validation, Data curation. **Abdo Hassoun:** Writing – review & editing, Validation, Data curation. **Zuhaib F. Bhat:** Writing – original draft, Validation, Supervision, Resources, Methodology, Data curation, Conceptualization.

## Declaration of competing interest

The authors declare that they have no known competing financial interests or personal relationships that could have appeared to influence the work reported in this paper.

## References

[1] Dutta D., Sit N. (2023). Application of natural extracts as active ingredient in biopolymer based packaging systems. J. Food Sci. Technol..

[2] Bhat Z.F., Bhat H.F., Manzoor M., Abdi G., Aadil R.M., Hassoun A., Aït-Kaddour A. (2024). Enhancing the lipid stability of foods of animal origin using edible packaging systems. Food Chem.: X.

[3] Alhujaili A., Nocella G., Macready A. (2023). Insects as food: consumers’ acceptance and marketing. Foods.

[4] Anwar R., Rabail R., Rakha A., Bryla M., Roszko M., Aadil R.M., Kieliszek M. (2022). Delving the Role of Caralluma fimbriata: An Edible Wild Plant to Mitigate the Biomarkers of Metabolic Syndrome. Oxid. Med. Cell. Longev..

[5] Lone A.B., Bhat H.F., Kumar S., Manzoor M., Hassoun A., Aït-Kaddour A., Mungure T.E., Muhammad Aadil R., Bhat Z.F. (2023). Improving microbial and lipid oxidative stability of cheddar cheese using cricket protein hydrolysates pre-treated with microwave and ultrasonication. Food Chem..

[6] Noor S., Kumar S., Bhat H.F., Hassoun A., Aadil R.M., Khandi S.A., Azad M.S., Abdi G., Bhat Z.F. (2024). Silkworm pupae protein-based film incorporated with Catharanthus roseus leaf extract-based nanoparticles enhanced the lipid stability and microbial quality of cheddar cheese. Food Hydrocoll. Health.

[7] Gao C., Zheng Y., Zhou R., Ma M. (2024). Active whey protein/hydroxypropyl methylcellulose edible films incorporated with cinnamaldehyde: Characterization, release kinetics and application to Mongolian cheese preservation. Int. J. Biol. Macromol..

[8] El-Sayed S.M., El-Sayed H.S., Hashim A.F., Youssef A.M. (2024). Valorization of edible films based on chitosan/hydroxyethyl cellulose/olive leaf extract and TiO2-NPs for preserving sour cream. Int. J. Biol. Macromol..

[9] Simmonds G., Woods A.T., Spence C. (2018). ‘Show me the goods’: Assessing the effectiveness of transparent packaging vs. product imagery on product evaluation. Food Qual. Prefer..

[10] Munir S., Hu Y., Liu Y., Xiong S. (2019). Enhanced properties of silver carp surimi-based edible films incorporated with pomegranate peel and grape seed extracts under acidic condition. Food Packag. Shelf Life.

[11] Silva S.P.M., Teixeira J.A., Silva C.C.G. (2023). Application of enterocin-whey films to reduce Listeria monocytogenes contamination on ripened cheese. Food Microbiol..

[12] Flórez M., Lopez-Sanchez P., Vázquez M., Cazón P. (2024). Sugar kelp Saccharina latissima extract as an innovative ingredient for chitosan films: Case study as cheese slice separators. Food Hydrocoll..

[13] Arif A., Sultan M.T., Nazir F., Ahmad K., Kashif M., Ahmad M.M., Shehzad F.K., Nazir M.A., Mushtaq S., Khalid M.U., Noman A.M., Raza H., Israr M., Sohail H., Rocha J.M. (2024). Exploring the therapeutic potential of *Caralluma fimbriata* for antioxidant and diabetes management: a 28-day rat model study. Toxicol. Res..

[14] Kandiah M., Chandrasekaran K.N. (2021). Green synthesis of silver nanoparticles using catharanthus roseus flower extracts and the determination of their antioxidant, antimicrobial, and photocatalytic activity. J. Nanotechnol..

[15] Shittu O.K., Stephen D.I., Kure A.H. (2017). Functionalization of biosynthesized gold nanoparticle from aqueous leaf extract of catharanthus roseus for antibacterial studies. Afr. J. Biomed. Res.

[16] Pande S.N., Bharati K.T., Wakchure S.K., Ghotekar S.K., Gujarathi D.B., Phatangare N.D. (2015). Green synthesis of silver nanoparticles by Caralluma fimbriata L. and its characterization. Indian J. Appl. Res..

[17] Haseena S., Shanavas S., Duraimurugan J., Ahamad T., Alshehri S.M., Acevedo R., Jayamani N. (2020). Study on photocatalytic and antibacterial properties of phase pure Fe_2_O_3_ nanostructures synthesized using Caralluma Fimbriata and Achyranthes Aspera leaves. Optik.

[18] Ghogare, P. B., & Aher, R. K. (2022). *Evaluation of Antimicrobial Efficacy of Caralluma adscendens var. fimbriata Extracts: A Molecular Docking Study*. www.ijcrt.org.

[19] Harsha L., Thangavelu L. (2017). Screening of ethanolic extracts of medicinal herbal drugs against oral microbes. Pharmacognosy J..

[20] Packialakshmi N., Naziya S. (2014). Green synthesis of silver nanoparticles from stem extracts of caralluma fimbriyata and its antibacterial activity. Internat. J. Appl. Sci. Biotechnol..

[21] Nourmohammadi A., Hassanzadazar H., Aminzare M., Hashemi M. (2023). The effects of whey protein/nanoclay biocomposite containing Thymus fedtschenkoi Ronniger essential oil and resveratrol on the shelf life of liqvan cheese during refrigerated storage. LWT.

[22] Jatav J., Chinchkar A.V., Bhattacharya B. (2023). Chitosan film with pineapple peel extract in the extension of shelf life of Indian cottage cheese: Release kinetics and bio-accessibility studies. Food Res. Int..

[23] Shafiei H., Saei-Dehkordi S., Moradi M., Molaei R. (2024). Preparation and antibacterial performance of bacterial nanocellulose sachet containing Zataria multiflora essential oil loaded halloysite nanotubes on Escherichia coli O157:H7 in cheese. LWT.

